# Supplementary material for: Molecular surveillance of resistance to pyrethroids insecticides in Colombian Aedes aegypti populations
Source: PLoS Negl Trop Dis. 2021 Dec 14;15(12):e0010001. doi: 10.1371/journal.pntd.0010001 (PMC8735628; doi:10.1371/journal.pntd.0010001)
Supplement: S1 Table — The HW equilibrium and the coefficient of endogamy (FIS) are shown. (DOCX) [file pntd.0010001.s001.docx]

**Supplementary Table S1.** Genotypic and allelic frequencies of the V410L, V1016I, and F1534C *kdr* alleles in Colombian mosquito populations. The HW equilibrium and the coefficient of endogamy (F_IS_) are shown.

| **Population** | **V410L** | | | | | | | | |
| --- | --- | --- | --- | --- | --- | --- | --- | --- | --- |
|  |  | **Allele frequency** | | **Genotype frequency** | | | **Hardy-Weinberg** | |  |
|  | **n** | **V** | **L** | **VV** | **VL** | **LL** | **χ^2^** | **p value** | **FIS** |
| Bello (1) | 55 | 0.95 | 0.05 | 50 | 5 | 0 | 0.125 | 0.724 | -0.048 |
| Itagüí (2) | 52 | 0.92 | 0.08 | 45 | 6 | 1 | 1.820 | 0.176 | 0.216 |
| Moniquirá (3) | 36 | 0.61 | 0.39 | 12 | 20 | 4 | 1.024 | 0.310 | -0.168 |
| Puerto Boyacá (4) | 38 | 0.51 | 0.49 | 11 | 17 | 10 | 0.419 | 0.520 | 0.105 |
| Puerto Bogotá (5) | 56 | 0.77 | 0.23 | 37 | 12 | 7 | 8.912 | 0.002 | 0.399 |
| Neiva (6) | 53 | 0.44 | 0.56 | 10 | 27 | 16 | 0.055 | 0.815 | -0.032 |
| Villavicencio (7) | 50 | 0.41 | 0.59 | 7 | 27 | 16 | 0.675 | 0.410 | -0.116 |
| Acacias (8) | 57 | 0.39 | 0.61 | 8 | 29 | 20 | 0.239 | 0.625 | -0.065 |
| Cúcuta (9) | 52 | 0.35 | 0.65 | 13 | 10 | 29 | 17.037 | 0.000 | 0.577 |
| Honda (10) | 53 | 0.71 | 0.29 | 28 | 19 | 6 | 0.948 | 0.330 | 0.134 |
| Acacias F7 pressure | 49 | 0.42 | 0.58 | 10 | 21 | 18 | 0.698 | 0.400 | 0.119 |
| Acacias F7 without pressure | 59 | 0.60 | 0.40 | 21 | 29 | 9 | 0.038 | 0.845 | -0.025 |
|  |  |  |  |  |  |  |  |  |  |
| **Population** | **V1016I** | | | | | | | | |
|  |  | **Allele frequency** | | **Genotype frequency** | | | **Hardy-Weinberg** | |  |
|  | **n** | **V** | **L** | **VV** | **VL** | **LL** | **χ^2^** | **p value** | **FIS** |
| Bello (1) | 59 | 0.94 | 0.06 | 52 | 7 | 0 | 0.235 | 0.628 | -0.063 |
| Itagüí (2) | 52 | 0.93 | 0.07 | 45 | 7 | 0 | 0.263 | 0.602 | -0.034 |
| Moniquirá (3) | 34 | 0.6 | 0.4 | 11 | 19 | 4 | 0.947 | 0.329 | -0.164 |
| Puerto Boyacá (4) | 40 | 0.64 | 0.36 | 11 | 29 | 0 | 13.012 | 0.000 | -0.573 |
| Puerto Bogotá (5) | 61 | 0.80 | 0.20 | 41 | 15 | 5 | 3.672 | 0.055 | 0.245 |
| Neiva (6) | 55 | 0.46 | 0.54 | 10 | 31 | 14 | 0.977 | 0.323 | -0.133 |
| Villavicencio (7) | 51 | 0.44 | 0.56 | 7 | 31 | 13 | 2.763 | 0.090 | -0.233 |
| Acacias (8) | 61 | 0.41 | 0.59 | 10 | 30 | 21 | 0.017 | 0.896 | -0.017 |
| Cúcuta (9) | 54 | 0.49 | 0.51 | 9 | 35 | 10 | 4.756 | 0.029 | -0.297 |
| Honda (10) | 54 | 0.70 | 0.30 | 28 | 20 | 6 | 0.675 | 0.411 | 0.112 |
| Acacias F7 pressure | 55 | 0.42 | 0.58 | 13 | 39 | 3 | 11.970 | 0.000 | -0.455 |
| Acacias F7 without pressure | 60 | 0.68 | 0.33 | 21 | 39 | 0 | 13.909 | 0.000 | -0.481 |
|  |  |  |  |  |  |  |  |  |  |
| **Population** | **F1534C** | | | | | | | | |
|  |  | **Allele frequency** | | **Genotype frequency** | | | **Hardy-Weinberg** | |  |
|  | **n** | **V** | **L** | **VV** | **VL** | **LL** | **χ^2^** | **p value** | **FIS** |
| Bello (1) | 60 | 0.45 | 0.55 | 12 | 30 | 18 | 0.006 | 0.937 | -0.010 |
| Itagüí (2) | 52 | 0.27 | 0.73 | 1 | 26 | 25 | 3.796 | 0.050 | -0.268 |
| Moniquirá (3) | 36 | 0.06 | 0.94 | 0 | 4 | 32 | 0.132 | 0.724 | 0.015 |
| Puerto Boyacá (4) | 40 | 0.00 | 1.00 | 0 | 0 | 40 | ND | ND | ND |
| Puerto Bogotá (5) | 62 | 0.22 | 0.78 | 2 | 23 | 37 | 0.491 | 0.483 | -0.089 |
| Neiva (6) | 55 | 0.05 | 0.95 | 0 | 6 | 49 | 0.183 | 0.668 | -0.058 |
| Villavicencio (7) | 51 | 0.06 | 0.94 | 1 | 4 | 46 | 4.339 | 0.037 | 0.292 |
| Acacias (8) | 59 | 0.08 | 0.92 | 0 | 9 | 50 | 0.402 | 0.525 | -0.083 |
| Cúcuta (9) | 53 | 0.00 | 1.00 | 0 | 0 | 53 | ND | ND | ND |
| Honda (10) | 53 | 0.08 | 0.92 | 0 | 9 | 44 | 0.456 | 0.499 | -0.093 |
| Acacias F7 pressure | 55 | 0.00 | 1.00 | 0 | 0 | 55 | ND | ND | ND |
| Acacias F7 without pressure | 60 | 0.00 | 1.00 | 0 | 0 | 60 | ND | ND | ND |

ND: non-determined
