# Supplementary material for: Molecular surveillance of resistance to pyrethroids insecticides in Colombian Aedes aegypti populations
Source: PLoS Negl Trop Dis. 2021 Dec 14;15(12):e0010001. doi: 10.1371/journal.pntd.0010001 (PMC8735628; doi:10.1371/journal.pntd.0010001)
Supplement: S2 Table — (DOCX) [file pntd.0010001.s002.docx]

**Supplementary Table S2**. Genotypes observed in Colombian *Aedes aegypti* populations and the association between genotypes frequencies and pyrethroid susceptibility profiles using the Spearman correlation test.

| **GENOTYPE** | Bello | Itagüí | Moniquirá | Puerto Boyacá | Puerto Bogotá | Neiva | Villavi- cencio | Acacias | Cúcuta | Honda | **TOTAL** | **Permethrin** | | **lambda-cyhalothrin** | |
| --- | --- | --- | --- | --- | --- | --- | --- | --- | --- | --- | --- | --- | --- | --- | --- |
|  |  |  |  |  |  |  |  |  |  |  |  | **Spearman** | | | |
|  |  |  |  |  |  |  |  |  |  |  |  | **r** | **p value** | **r** | **p value** |
| VV_410_/VV_1016_/FF_1534_ | 10 | 1 | 0 | 0 | 2 | 0 | 0 | 0 | 0 | 0 | **13** | 0.098 | 0.789 | -0.723 | 0.018 |
| VV_410_/VV_1016_/CC_1534_ | 13 | 18 | 7 | 11 | 14 | 9 | 6 | 5 | 5 | 23 | **111** | -0.505 | 0.137 | -0.571 | 0.084 |
| VV_410_/VV_1016_/FC_1534_ | 24 | 26 | 3 | 0 | 18 | 1 | 1 | 3 | 0 | 5 | **81** | -0.42 | 0.227 | -0.756 | 0.011 |
| LL_410_/VV_1016_/CC_1534_ | 0 | 0 | 0 | 0 | 0 | 0 | 0 | 0 | 3 | 0 | **3** | 0.525 | 0.119 | 0.406 | 0.244 |
| VL_410_/VV_1016_/CC_1534_ | 0 | 0 | 1 | 0 | 0 | 0 | 0 | 0 | 1 | 0 | **2** | 0.209 | 0.563 | 0.035 | 0.924 |
| LL _410_/II_1016_/CC_1534_ | 0 | 0 | 3 | 0 | 5 | 12 | 12 | 19 | 8 | 6 | **65** | -0.006 | 0.986 | 0.739 | 0.015 |
| VL_410_/II_1016_/CC_1534_ | 0 | 0 | 1 | 0 | 0 | 0 | 0 | 0 | 2 | 0 | **3** | 0.313 | 0.378 | 0.138 | 0.703 |
| VV_410_/VI_1016_/CC_1534_ | 0 | 0 | 0 | 0 | 1 | 0 | 0 | 0 | 8 | 0 | **9** | 0.392 | 0.263 | 0.221 | 0.548 |
| VV_410_/VI_1016_/FC_1534_ | 1 | 0 | 1 | 0 | 0 | 0 | 0 | 0 | 0 | 0 | **2** | 0.131 | 0.719 | -0.588 | 0.074 |
| LL_410_/VI_1016_/CC_1534_ | 0 | 1 | 1 | 10 | 0 | 4 | 3 | 1 | 8 | 0 | **28** | 0.337 | 0.340 | 0.474 | 0.167 |
| LL_410_/VI_1016_/FC_1534_ | 0 | 0 | 0 | 0 | 2 | 0 | 0 | 0 | 0 | 0 | **2** | -0.058 | 0.873 | -0.174 | 0.631 |
| VL_410_/VI_1016_/FF_1534_ | 0 | 0 | 0 | 0 | 0 | 0 | 1 | 0 | 0 | 0 | **1** | 0.234 | 0.516 | 0.290 | 0.416 |
| VL_410_/VI_1016_/CC_1534_ | 3 | 6 | 17 | 17 | 11 | 22 | 23 | 21 | 7 | 14 | **141** | -0.152 | 0.674 | 0.345 | 0.328 |
| VL_410_/VI_1016_/FC_1534_ | 2 | 0 | 0 | 0 | 1 | 5 | 3 | 6 | 0 | 4 | **21** | -0.258 | 0.472 | 0.313 | 0.379 |
| **TOTAL** | **53** | **52** | **34** | **38** | **54** | **53** | **49** | **55** | **42** | **52** | **482** |  |  |  |  |

Significant correlations (p <0.05) after Bonferroni test correction are shaded in gray
