# Supplementary material for: Molecular surveillance of resistance to pyrethroids insecticides in Colombian Aedes aegypti populations
Source: PLoS Negl Trop Dis. 2021 Dec 14;15(12):e0010001. doi: 10.1371/journal.pntd.0010001 (PMC8735628; doi:10.1371/journal.pntd.0010001)
Supplement: S3 Table — P-values were calculated using the Kruskal-Wallis test for multiple comparisons. (DOCX) [file pntd.0010001.s003.docx]

**Supplementary Table S3**. Mean of enzyme activities for Acetylcholinesterase (AChE), glutathione-S-transferases (GST), β-esterases (β-EST), Mixed function oxidases (MFO), and α-esterases (α-EST), detected in *Aedes aegypti* from Acacías with and without insecticide pressure. P-values were calculated using the Kruskal-Wallis test for multiple comparisons.

| **Enzyme** | **Susceptible reference strain (N=90)** | **Acacías pressure (N=40)** | **Acacías without pressure (N=40)** | ***p-value*** |
| --- | --- | --- | --- | --- |
|  | **Mean ± SD** | **Mean ± SD** | **Mean ± SD** |  |
| AChE | 26.92 ± 5.208 | 37.92 ± 12.93 | 28.56 ± 9.092 | <0.0001 |
| GST | 0.3408 ± 0.1475 | 0.5076 ± 0.1416 | 0.4074 ± 0.1504 | <0.0001 |
| β-EST | 8.458 ± 3.308 | 8.482 ± 2.171 | 10.94 ± 3.016 | <0.0001 |
| MFO | 40.67 ± 13.97 | 57.76 ± 13.74 | 50.92 ± 16.84 | <0.0001 |
| α-EST | 8.934 ± 3.128 | 9.694 ± 1.773 | 10.36 ± 2.534 | 0.0005 |
